# Biomimetic Origami-Based Soft Robotic Grippers with Two-Stage Grasping

**DOI:** 10.3390/biomimetics11070466

**Published:** 2026-07-03

**Authors:** Ana Botrić, Goran Gregov

**Affiliations:** University of Rijeka, Faculty of Engineering, Vukovarska 58, 51000 Rijeka, Croatia; abotric@uniri.hr

**Keywords:** biomimetics, origami, soft robotics, robotic gripper, two-stage grasping

## Abstract

This study presents the innovative design and development of biomimetic origami-based soft robotic grippers capable of two-stage grasping. Inspired by the biological structure of the sea urchin mouth, which combines external rigid teeth with an internal soft membrane, the proposed grippers employ origami architectures to achieve coordinated two-stage grasping. Novel waterbomb and Miura-ori origami architectures were introduced, enabling the formation of external and internal teeth. The developed grippers integrate an elastomeric membrane with an internal origami structure that enables contraction-driven folding under negative-pressure actuation. Multiple gripper configurations with varying dimensions are fabricated using paper and polymer-laminated paper skeletons. An energy-based modeling framework is introduced to describe the pressure–force relationship while accounting for the effects of structural deformation. Experimental evaluations conducted at different negative-pressure values quantified grasping performance and holding force. Imprint-based analysis confirmed the two-stage grasping mechanism, while grasping capability investigations demonstrated compliant interaction with delicate objects. Holding forces were measured using cylindrical metal and spherical wooden test objects of varying sizes and orientations. The waterbomb-based gripper achieved the most consistent performance, particularly for cylindrical objects, reaching a maximum holding force of 70 N, whereas the Miura-ori provided improved adaptability and higher holding forces for spherical objects, reaching 74.8 N, and maximum force-to-weight ratios of 327.2 and 346.6 were achieved for the waterbomb- and Miura-ori-based grippers, respectively.

## 1. Introduction

Origami is the ancient Japanese art of paper folding, originally developed for wrapping gifts and fans, which held significant symbolic value. Origami butterflies (*Mecho* and *Ocho*) are among the earliest symbolic forms, used to decorate sake bottles at wedding ceremonies, predating the origami crane (*Orizuru*), which is today the most iconic origami art and symbolizes healing, peace and good fortune [1]. Nowadays, the principles of origami are applied far beyond their original cultural context, encompassing fields such as architecture, furniture design, fashion, medical technology and robotics [2,3,4,5].

Soft robotics represents a fundamental shift from conventional rigid-body robotics, employing compliant materials and distributed actuation to enable adaptive, resilient, and safe interaction with humans [6,7,8]. Among actuation approaches in soft robotics, pneumatic actuation [9,10] is widely adopted due to its compatibility with compliant materials and its ability to provide precise multidirectional motion within low-complexity pressure control architectures [11,12,13]. A pneumatic soft robot can operate under positive and/or negative pressure. Positive-pressure actuation increasing the risk of material rupture, whereas negative-pressure actuation is inherently limited to a maximum pressure differential of 1 bar [14,15,16].

Pneumatic soft robotics has found one of its most widespread applications in soft robotic grippers, which enable safe and adaptive grasping of objects with varying shapes, sizes, and stiffness [17,18]. Recent studies have explored a variety of soft gripper designs, including origami-based enclosing grippers [19], insect-inspired claw mechanisms [20], and stiffness-tunable soft grippers with enhanced load-carrying capacity [21]. Their functionality relies on deformable chambers that bend, twist, or contract under pressure actuation and can be fabricated from various compliant materials [22,23]. When combined with origami principles, soft robotic grippers achieve enhanced performance because fold-based geometries enable anisotropic deformation, higher force-to-weight ratios and improved compactness through deployable structures. Origami-based soft robots most commonly employ negative-pressure actuation [24], whereas positive-pressure actuation is more often applied in kirigami-based robotics [25].

The application of biological principles in soft robotics has led to innovative design approaches that enhance adaptability, efficiency, and functionality [26]. In particular, biomimetic approaches transform biological mechanisms into engineering solutions by reproducing their underlying geometric and kinematic principles [27]. In a study [28], a biomimetic origami-based soft robotic gripper was proposed, drawing inspiration from leech locomotion, characterized by cyclic alternation between elongation and bending, upon which an origami skeleton was designed and actuated using negative pressure. The results demonstrate that the gripper is capable of grasping objects with diverse shapes, sizes, weights, and surface textures. Soft robotic grippers presented in [29] employ a dual-origami design based on a Miura-ori polyhedron and a zig-zag folded Miura-ori pattern, enabling large shape deformations and bending.

In [30], a soft robotic gripper based on a modified waterbomb origami pattern was proposed to achieve stable and repeatable grasping with nearly constant gripping force under dynamic operating conditions. The gripper is fabricated using 3D printing, employing TPU and PLA materials. A study [31] made a significant contribution to the development of origami-based soft robotic grippers. The proposed design employs the waterbomb-based origami “Magic Ball” structure, enabling radial volume contraction greater than 90%. Three prototypes were developed and experimentally evaluated using different skeleton material combinations (PET-PVC-Kapton, Dragon Skin 30, and Dragon Skin 50).

In this study, a biomimetic soft robotic gripper is designed and fabricated, drawing inspiration from the biomechanical principles of the sea urchin mouth and leveraging origami-based engineering design. Observations of the sea urchin’s (*Echinoidea*) feeding mechanism during diving reveal that its mouth operates as a two-stage grasping mechanism, combining synchronized rigid external teeth and a compliant internal membrane to enable efficient and adaptive food capture and feeding [32,33]. To translate this concept into a pneumatic robotic gripper, an origami-based design approach is adopted, providing a simple, low-cost structure with two functional grasping stages. The elementary gripper design is based on a hermetically sealed elastomeric membrane and an internal origami structure, known as the skeleton, which defines the gripper’s shape and kinematics. When negative pressure is applied, the membrane contracts and induces folding of the origami skeleton, thereby enabling grasping. Investigation of origami patterns shows that the waterbomb and Miura-ori patterns enable the formation of two distinct stages corresponding to external and internal teeth, allowing synchronized motion and controlled opening and closing of the grippers. For detailed analysis, origami skeletons were fabricated in two-dimensional variations and from different materials, including paper and polymer-laminated paper. Extensive experimental investigations were conducted to evaluate the grasping capability of the developed grippers using both modeling clay and real objects under varying negative pressure. Maximum holding force tests were performed for cylindrical and spherical test objects of different sizes and orientations at different negative pressures.

**Figure 1:**
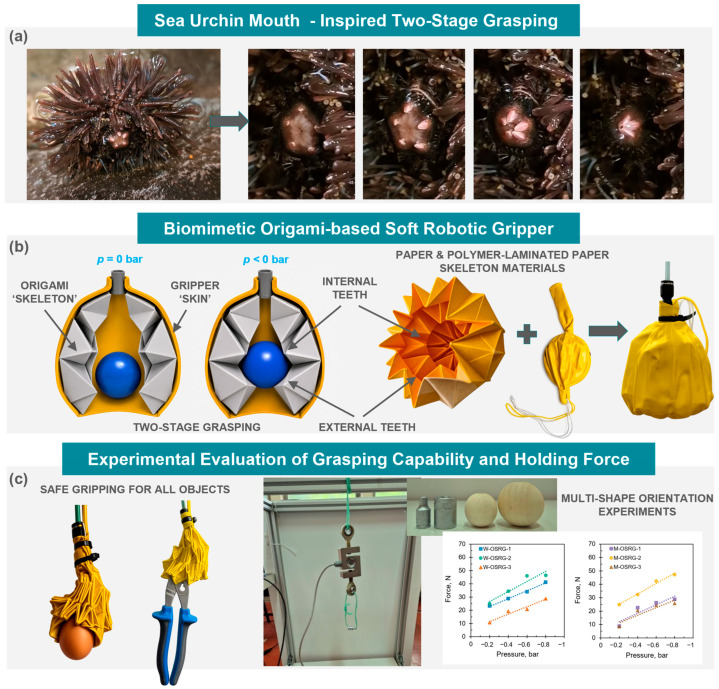
Origami-based soft robotic gripper: (**a**) Biomimetic two-stage grasping inspired by the sea urchin mouth; (**b**) Design, development and working principle; (**c**) Laboratory setup and experimental evaluation of holding force for WH12 in horizontal direction.

The design approach used in this research is summarized graphically in Figure 1, and the main contributions of this work are as follows:

- Introduction of an innovative biomimetic origami-based soft robotic gripper inspired by the sea urchin mouth, integrating the functions of external rigid teeth and an internal soft membrane to achieve two-stage grasping;
- Development of novel waterbomb and Miura-ori origami architectures featuring geometrically programmed external and internal teeth that enable coordinated two-stage grasping;
- Fabrication, and experimental evaluation of multiple gripper configurations with varying dimensions and skeleton materials, including paper-based and polymer-laminated paper structures;
- Comprehensive experimental assessment of grasping capability and holding force using delicate, cylindrical, and spherical test objects under different negative-pressure conditions;
- Demonstration of high normalized grasping performance, with force-to-weight ratios reaching 346.6, confirming the effectiveness of the developed grippers.

The remainder of the paper is organized as follows. Section 2 describes the design and development of a biomimetic origami-based soft robotic gripper with two-stage grasping and introduces an energy-based model to characterize the pressure–force relationship. Section 3 presents the experimental results, evaluating the grasping performance and the holding force under varying negative pressure. Finally, Section 4 summarizes the main conclusions.

## 2. Materials and Methods

This section presents the design and development of a biomimetic soft robotic gripper with two-stage grasping, inspired by the biomechanical structure of the sea urchin mouth. The gripper is based on waterbomb and Miura-ori origami architectures enabling two-stage grasping and fabricated with varying dimensions and materials. An energy-based model is further introduced to describe the pressure–force relationship of the developed grippers.

### 2.1. Design and Development of a Biomimetic Origami-Based Soft Robotic Gripper with Two-Stage Grasping

A detailed analysis of the sea urchin’s mouth, also known as Aristotle’s lantern (*Lanterna Aristotelis*), revealed a biological mechanism that combines synchronized motion with high adaptability for grasping objects of varying shapes. The sea urchin’s mouth consists of external, interconnected rigid teeth (*Maxilla*) that coordinate their opening and closing to scrape food from rocky substrates and capture food particles. In addition to the external teeth, the sea urchin mouth contains internal soft tissue associated with the peristomial membrane (*Membrana peristomialis*), which facilitates the collection and transport of food toward the digestive tract (see Figure 1a). Therefore, the sea urchin mouth can be interpreted as a two-stage grasping system composed of external and internal teeth that operate synchronously to enable food capture.

**Figure 2:**
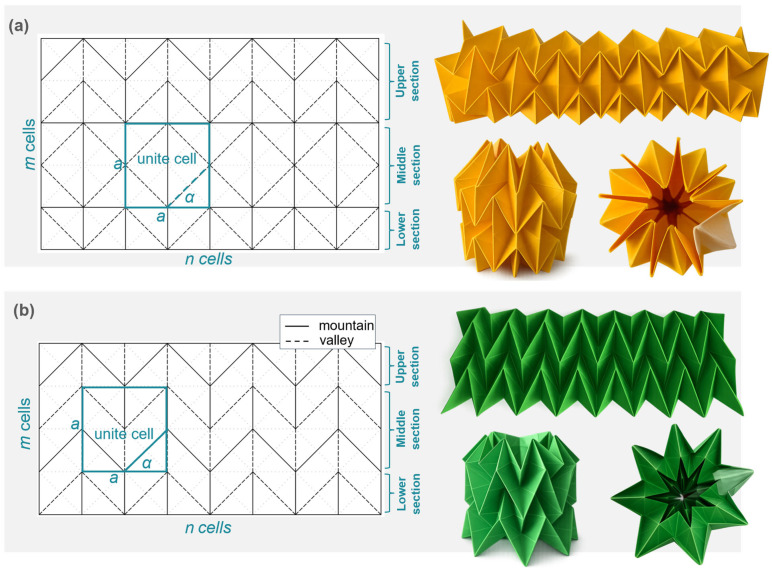
Origami pattern, folded configuration, transformation into a gripper skeleton, and bottom view of the origami skeleton showing the internal and external teeth: (**a**) Waterbomb (yellow); (**b**) Miura-ori (green).

This biological principle inspires the design of a soft robotic gripper composed of two coordinated, tooth-like segments that enable effective grasping. To translate this concept into a soft robotic gripper, an origami-based approach is assumed, offering simple and low-cost fabrication. The origami-based soft robotic gripper (OSRG) is actuated by negative pressure and comprises two main components: an origami skeleton defining the geometry and kinematics, and a flexible outer membrane (“skin”) that contracts under negative pressure and transfers motion to the skeleton (see Figure 1b). Geometric analysis of the origami patterns used for the OSRG skeleton shows that the waterbomb and Miura-ori patterns enable the formation of two distinct segments corresponding to external and internal teeth. As shown in Figure 1b and Figure 2, the waterbomb and Miura-ori skeletons consist of external and internal teeth that move synchronously, enabling controlled opening/closing and grasping of an object.

Origami patterns are defined by three fundamental elements: fold lines, vertices where folds intersect, and facets bounded by these folds, with folds further classified as mountain (convex) or valley (concave) creases. Collectively, these parameters define the unit cell, whose periodic repetition (tessellation) results in complex origami structures. The waterbomb pattern formed by tessellating the unit cell, where solid and dashed lines denote mountain and valley creases, respectively (Figure 2a). To achieve a two-stage tooth configuration, we introduce a modified waterbomb origami pattern that enables a novel gripper architecture. A half-height unit cell in the lower section forms the external teeth, while a standard waterbomb cell in the middle of the pattern creates the internal teeth. A corresponding folding approach in the upper section produces a conical structure (Magic-Ball design), improving the gripper’s geometric and kinematic performance. After folding, the initially flat waterbomb structure transforms (rolled) into an origami gripper skeleton, as illustrated in Figure 2a.

Figure 2b illustrates the Miura-ori pattern and its main geometric parameters, which, upon folding, transform from an initially flat structure into the gripper skeleton. To achieve a two-stage tooth configuration, a modified Miura-ori origami pattern is introduced, where a half-height unit cell in the lower section forms the external teeth, a standard Miura-ori cell in the middle section generates the internal teeth, and a half-height unit cell in the upper section creates a conical structure. The developed Miura-ori origami architecture generates a slightly conical inner skeleton geometry that promotes sequential two-stage grasping, where contact with the object is first established by the internal teeth and subsequently reinforced by the external teeth.

The skin of the OSRG was fabricated using a commercially available latex balloon with a thickness of 150 μm (see Figure 1b). Due to its high elasticity, the latex balloon precisely fits the origami skeleton, enveloping it securely and enabling uniform contact with objects. An additional advantage of the selected balloon is the factory-formed loop at its apex, which serves to transmit forces from the gripper to the robotic arm. A non-stretchable rope is threaded through the loop, and after the origami skeleton is inserted into the balloon, the loop and rope are pulled through the origami skeleton, securing tight adhesion between the balloon and the inner side of the skeleton. The balloon opening is cut and fitted with a standard pneumatic connector, and the assembly is secured using zip ties to ensure airtight sealing.

Determining the optimal dimensions of the origami skeletons proved challenging, as they must allow insertion into the latex balloon while minimizing gaps. To ensure a consistent comparison, unit cell dimensions were selected to be similar for both origami patterns (Figure 2). A square unit cell, *a* = 36 mm, was selected based on preliminary trials (*a* = 20, 30, and 40 mm). Initial experimental observations indicated that the OSRG maintains its functionality even with increased gaps between the skeleton and the balloon. Accordingly, two variants (OSRG-1 and OSRG-2) were developed for each pattern, differing in the number of columns (*n*), with variant 1 using *n* = 8 and variant 2 using *n* = 10. The height of the origami skeleton is a key design parameter, ensuring that the balloon loop can pass through the structure while maintaining reliable contact with its inner surface. It is defined by the number of rows (*m*) in the origami pattern and was determined through iterative adjustment. The optimal height was determined to be *m* = 2.5 for both waterbomb variants (W-OSRG-1 and W-OSRG-2) and *m* = 2 for both Miura-ori variants (M-OSRG-1 and M-OSRG-2).

The origami skeletons of both gripper versions were fabricated by manually folding 190 g/m^2^ paper, owing to its favorable formability and adequate durability. Upon transformation into a skeleton, the edges of the flat origami structure are joined. For edge joining, several types of adhesive tapes were tested, with 25 mm-wide 3M Micropore surgical tape (3M Company, Maplewood, MN, USA) proving to be the most effective. Owing to its elasticity and paper-like texture, it allowed folding without significantly affecting the deformation of the origami skeleton. In contrast, PVC adhesive tape significantly increases the local stiffness at the joints, thereby altering the deformation behavior.

Preliminary experimental tests with the paper origami skeletons revealed deformation of the skeleton during repeated actuation. To enhance durability and prolong lifespan, the paper was reinforced with a 100 μm thick transparent self-adhesive polymer film. The self-adhesive film was then applied to both sides of the paper, ensuring that the edges were firmly pressed to prevent delamination. This treatment enhances durability under repeated loading cycles while maintaining the flexibility and compliant characteristics of the OSRG. Polymer-laminated paper origami skeletons were additionally fabricated for the larger dimension variants of both origami architectures, denoted as variant 3 (W-OSRG-3 and M-OSRG-3), for further experimental analysis.

### 2.2. Energy-Based Modeling of the Gripper Pressure–Force Relationship

An energy-based modeling framework describes gripper behavior through simplified relationships between measurable variables, while retaining the dominant effects of the underlying physical mechanisms. In this context, the objective of the proposed model is not to fully resolve the complex nonlinear mechanics of the OSRG, but to establish an analytical description that captures its response under negative-pressure actuation. The formulation enables a physically grounded interpretation of the system behavior and allows direct qualitative and semi-quantitative comparison with experimental measurements. At the same time, it retains the essential coupling between pressure-induced deformation and structural response through a set of simplifying assumptions. To this end, the model is expressed in a system-level reduced-order form, enabling direct comparison with experimentally measured holding forces. The resulting formulation should therefore be interpreted as a physics-informed phenomenological model that captures the dominant trends of the system, rather than a fully predictive continuum description. This approach provides a compact and physically interpretable representation of the pressure–force relationship across different origami configurations. The origami pattern geometry is parameterized by the set

(1) *p* = {*a*,*α*,*m*,*n*},

where *a* denotes the dimensions of the square unit cell, *α* is the folding angle of the origami unit cell, and *m* and *n* represent the number of unit cells in the axial and horizontal (circumferential) directions (see Figure 2). While the full kinematics of the origami structure involves multiple coupled degrees of freedom, experimental observations indicate that the deformation is predominantly governed by a symmetric folding mode. This behavior is characteristic of origami structures, where the global response is often dominated by a small number of kinematically admissible deformation modes. Consequently, the system can be approximated as evolving along a low-dimensional manifold, which can be parameterized by a single generalized coordinate, *q* = *θ*. Here, *θ* represents the global folding angle and uniquely determines the macroscopic geometry of the structure [34]. This assumption enables a reduced-order description that captures dominant behavior while neglecting higher-order deformation modes, consistent with the experimentally observed uniform and axisymmetric deformation. Under this assumption, the axial length and circumference are approximated as:

(2) $$L\left(\theta \right)=m\;l_{z}\left(\theta \right),C\left(\theta \right)=n\;l_{c}\left(\theta \right).$$

where *l*_z_(*θ*) and *l*_c_(*θ*) are the effective axial and circumferential projections of a single origami unit cell. These projections can be expressed as summarized in Table 1 for waterbomb and Miura-ori origami. The effective radius and diameter of the tubular origami structure are as follows:

(3) $$R(\theta )=\frac{C(\theta )}{2\pi }D(\theta )=2R(\theta )$$

The deformation of the gripper arises from the interaction between negative pressure, origami crease elasticity, membrane elasticity, and contact with the grasped object. To capture this behavior in a tractable manner, a reduced-order, energy-based formulation is adopted, in which the dominant mechanical contributions are represented through a single generalized coordinate. Within this framework, the enclosed volume of the structure is approximated as:

(4) $$V(\theta )=\chi \pi R^{2}(\theta )L(\theta ),$$

where $\left.\chi \in (0,1\right]$ is a shape correction factor accounting for deviations from an ideal cylindrical geometry caused by the faceted origami structure and the partially conical geometry of the gripper. Although this approximation introduces a modeling error, it significantly simplifies the analytical treatment while preserving the dominant dependence of the volume on the folding kinematics. Within the considered operating range, χ is treated as an effective constant parameter and is identified from experimental measurements. OSRG actuation is driven by a pressure differential (Δ*p*) between the atmosphere and the gripper’s inside, which is incorporated into the model through a pressure-induced potential energy term:

(5) $$U_{p}\left(\theta \right)=-\Delta p\;V\left(\theta \right).$$

The deformation of the origami structure is primarily localized along the crease lines, which are modeled as torsional springs. The strain energy associated with the crease deformation is expressed as:

(6) $$U_{o}(\theta )=\frac{1}{2}k_{\theta }(\theta -\theta_{0}{)}^{2}+\frac{1}{4}k_{3}(\theta -\theta_{0}{)}^{4},$$

where *k*_θ_ is the effective linear stiffness associated with the crease pattern, *k*_3_ accounts for nonlinear stiffening, and *θ*_0_ denotes the natural folding angle. The inclusion of the higher-order term enables the model to capture the increased resistance observed at larger deformations. In addition to the origami skeleton, the enclosing elastomeric membrane (latex balloon) contributes to the overall mechanical response by storing elastic strain energy during deformation. The corresponding energy contribution is expressed as:

(7) $$U_{m}(\theta )=\frac{1}{2}k_{m}(\theta -\theta_{m}{)}^{2},$$

where *k*_m_ represents the effective stiffness of the membrane and *θ*_m_ denotes its preferred configuration, i.e., the folding angle at which the membrane energy is minimized. This lumped representation provides an effective description of the membrane response, capturing its dominant contribution while implicitly incorporating its continuum mechanics.

During grasping, the gripper establishes contact with the object of diameter *d*_o_. Contact is assumed to occur when the gripper diameter satisfies *D*(*θ*) ≤ *d*_0_ and the resulting interaction is described using a penetration-based formulation. The penetration depth is:

(8) $$\delta (\theta )=max(0,d_{o}-D(\theta )),$$

and the corresponding contact energy is given by:

(9) $$U_{c}(\theta )=\frac{1}{2}k_{c}\delta^{2}(\theta ),$$

where *k*_c_ is the effective contact stiffness. This penalty-based formulation provides a smooth and computationally efficient description of the contact interaction, capturing its dominant response at the system level. The analysis is restricted to quasi-static conditions, assuming negligible dynamic effects during actuation. The overall behavior of the system is governed by the total potential energy:

(10) $$\Pi (\theta )=U_{o}(\theta )+U_{m}(\theta )+U_{c}(\theta )-\Delta pV(\theta ).$$

The equilibrium configuration is determined by the stationarity condition dΠ/d*θ* = 0, which reflects the balance between elastic resistance and pressure-induced volume change. To quantify the mechanical interaction between the gripper and the object, the radial grasping force is derived using the principle of virtual work. This establishes a direct relationship between the energy-based formulation and the force transmitted at the contact interface, linking variations of the generalized coordinate *θ* to the resulting radial deformation. The grasping force can be expressed as:

(11) $$F_{g}(\theta )=\frac{1}{|J_{D}(\theta )|}\left(\Delta p\frac{dV}{d\theta }-\frac{dU_{o}}{d\theta }-\frac{dU_{m}}{d\theta }\right),$$

where *J*_D_(*θ*) = d*D*/D*θ* denotes the kinematic Jacobian relating the folding coordinate to the radial deformation of the gripper. In the contact regime, a simplified approximation is introduced by relating the grasping force to the penetration depth:

(12) $$F_{g}\left(\theta \right)\approx k_{c}\;\delta \left(\theta \right).$$

This expression provides a practical estimate of the contact force while maintaining consistency with the underlying energy-based formulation. This approximation is consistent with the penalty-based contact formulation, where the contact force corresponds to the derivative of the contact energy with respect to the penetration depth. The total holding force acting on the object is then expressed as:

(13) $$F_{h}=\mu F_{g}+F_{\mathrm{geo}},$$

where *μ* is the friction coefficient and *F*_geo_ accounts for the additional contribution due to geometric enclosure. This formulation highlights the combined role of frictional and geometric effects in determining the overall grasping performance. To enable direct comparison with experimentally measured holding forces, the energy-based formulation is expressed at the system level by mapping the internal variables to the applied negative pressure. Since quantities such as the folding angle *θ*, penetration depth *δ*, and force *F*_geo_ are not directly measurable, the model is reformulated in terms of the externally controlled pressure. This transformation can be interpreted as a projection of the underlying physics-based model onto experimentally accessible variables. Within the considered operating range, the resulting nonlinear pressure–force relationship can be represented using a quadratic approximation:

(14) $$F_{h}\left(\Delta p\right)=F_{0}+K_{1}\Delta p+K_{2}\Delta p^{2},$$

where *F*_0_, *K*_1_, and *K*_2_ are effective lumped parameters. *F*_0_ represents a baseline contribution associated with initial geometric confinement, membrane pretension, and the geometric component *F*_geo_. The coefficient *K*_1_ characterizes the linear sensitivity of the holding force to negative pressure and reflects the combined influence of geometric and material parameters (*a*, *α*, *m*, *n*, *k*_θ_, *k*_m_, *χ* and *μ*). The coefficient *K*_2_ captures the nonlinear response at higher pressure levels, arising from nonlinear stiffness (*k*_3_), contact effects (*k*_c_), and geometric nonlinearities in the kinematic relations *V*(*θ*) and *D*(*θ*). Comparison with the experimental results presented in Section 3.2 confirms that the proposed framework captures the dominant pressure–force trends with good quantitative agreement over the considered operating range. By linking energy-based modeling to experimentally measurable performance through a system-level representation, the framework reveals the coupled roles of geometry, material properties, and contact mechanics in object grasping.

## 3. Experimental Results and Discussion

Experimental assessments were conducted at the Hydraulic and Pneumatic Laboratory at the University of Rijeka, Faculty of Engineering, using the following pneumatic equipment: a CECCATO CSM 7.5 compressor with an air preparation unit (Ceccato, Grisignano di Zocco, Italy), a FESTO VPPI-5L-G18-1V1H-V1-S1D proportional pressure control valve, and a FESTO VN-05-H-T2-PQ1-VQ1-RQ vacuum ejector (Festo SE & Co. KG, Esslingen am Neckar, Germany). For data collection and processing, measurement equipment including the National Instruments NI myRIO 1900 device (National Instruments Corporation, Austin, TX, USA) and a Zemic H3G-C3-50kg-6B load cell (Zemic Europe B.V., Breda, The Netherlands) were used. Two experimental assessments were performed under different negative-pressure values: the first focused on evaluating the grasping capability of the developed grippers for objects with varying geometries and fragilities, whereas the second quantitatively assessed the holding force under different grasping conditions.

### 3.1. Experimental Evaluation of Grasping Capability

**Figure 3:**
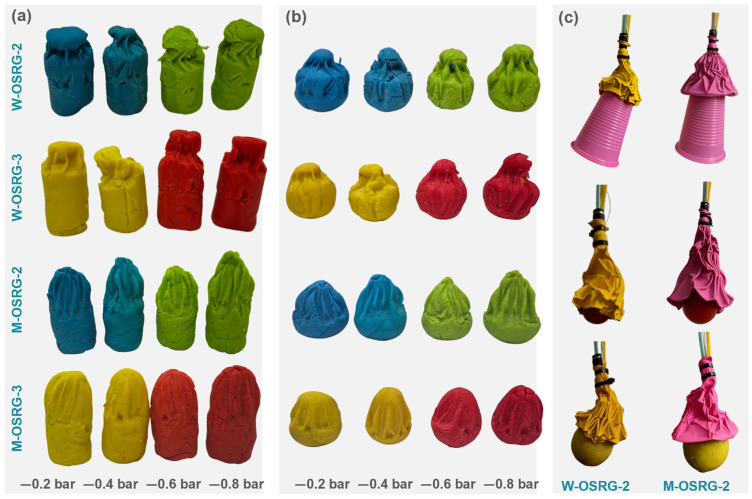
Imprints produced on test objects by the developed OSRG under varying negative pressures: (**a**) CL1; (**b**) CL2; (**c**) Experimental evaluation of grasping performance of W-OSRG-2 (yellow) and M-OSRG-2 (pink) on a plastic cup, cherry tomato and lemon.

This experimental evaluation aimed to characterize the contact area formed by the developed OSRG and to evaluate their capability for two-stage grasping and soft interaction during the manipulation of delicate objects. The test objects were fabricated from modeling clay Play-Doh (Hasbro, Pawtucket, RI, USA) in cylindrical (CL1) and spherical (CL2) forms; their geometrical and physical characteristics are provided in Table 2. The deformation of the test objects was induced using OSRG-2 and OSRG-3, which have identical dimensions but differ in the material composition of the skeleton. Experiments were conducted at negative-pressure values of −0.2, −0.4, −0.6, and −0.8 bar. The interaction between the grippers and the test object surfaces resulted in characteristic imprints, enabling visualization of the grasp geometry and contact area, as shown in Figure 3. Both the shape and intensity of the imprints depend strongly on the applied negative pressure: increasing pressure values lead to higher grasping forces and, consequently, more deformation of the test objects.

Analysis of the imprints on the cylindrical test objects produced by the W-OSRG reveals pronounced local deformations in the form of tooth-like features at two distinct heights, confirming the two-stage grasping mechanism (Figure 3a). The obtained imprints indicate that the internal teeth generate higher grasping forces, resulting in larger imprint areas. This behavior can be attributed to the sequential establishment of contact, first by the internal teeth and subsequently by the external teeth, enabled by the geometry of the skeleton’s upper section. At higher negative-pressure values, significant deformation of the test objects is observed, in some cases approaching near splitting. A similar grasping behavior is observed for W-OSRG-2 and W-OSRG-3; however, the paper-based version consistently achieves higher test object deformation, i.e., grasping forces. This difference arises from the higher strain energy of the polymer-laminated paper origami skeleton, leading to a reduction in the effective grasping force transmitted to the object.

Analysis of the imprints produced by the M-OSRG on the cylindrical test object confirms a two-stage grasping mechanism enabled by the conical groove geometry (Figure 3a). Due to the slight conical geometry of the inner side of the Miura-ori skeleton, contact is first established by the internal teeth and subsequently by the external teeth. However, at the final stage of deformation, the imprints of the internal and external teeth tend to overlap, making the two-stage contact less visually distinct, although indentations from the external teeth can still be observed on the side of the test object. M-OSRG-2 with a paper skeleton exhibits greater deformation and higher grasping forces than the polymer-laminated paper M-OSRG-3, owing to reduced strain energy, consistent with the waterbomb gripper. Analysis of the imprints on the spherical test objects (Figure 3b) reveals patterns similar to those observed on the cylindrical objects, with the imprints of the external teeth being more pronounced.

Furthermore, stable grasping performance was experimentally evaluated on delicate objects, including a plastic cup, a cherry tomato, and a lemon, as well as on industrial objects such as pliers, a pneumatic throttle check valve, and an aluminum angle bracket (Table 2). All tests were conducted using second variant of the developed grippers: W-OSRG-2 (yellow) and M-OSRG-2 (pink). Special attention was given to achieving a stable and gentle grasp at the lowest possible negative pressure (−0.1 bar) sufficient to lift each object, while avoiding visible surface damage or structural deformation. Figure 3c illustrates the successful grasping of a plastic cup, a cherry tomato and a lemon, demonstrating that all developed grippers provided stable and gentle handling without causing any visible damage or deformation. Moreover, the results confirm reliable grasping of irregularly shaped items, with stable grasps achieved across all tests and the ability to handle heavier objects such as pliers (see Figure 1c). The grasping capability of the developed grippers for the mentioned test objects is provided in Supplementary Materials (Video S1).

Based on the experimental results, it can be concluded that the proposed two-stage grasping mechanism is governed by the geometric arrangement of the internal and external teeth, enabling sequential object engagement and stable grasp formation. The relative contribution of each grasping stage depends on the geometry and dimensions of the grasped object, as confirmed by imprint analysis experiments conducted on objects of different shapes and sizes. However, a detailed quantification of the contact force distribution and the individual contribution of each grasping stage was beyond the scope of the present study.

### 3.2. Experimental Evaluation of Holding Force

The following experiments were conducted to quantify the holding force of the developed OSRG at negative-pressure values of −0.2, −0.4, −0.6, and −0.8 bar. Measurements were performed using a frame assembled from aluminum strut profiles, with the load cell suspended by a non-stretchable polypropylene rope, while the test object was attached to the opposite side of the load cell using the same type of rope (see Figure 1c). The gripper first grasped the test object at a specified negative pressure, after which the object was manually pulled downward along the gripper axis until slippage occurred. The maximum holding force was recorded at the onset of slippage using the load cell. For each gripper configuration and test object, five repeated measurements were performed. As the experiments involved manual pulling of the gripper, the results are subject to human variability, including variations in pulling speed and minor deviations from the ideal gripper axis. Accordingly, the standard deviation of the holding force was calculated for all measurements. The resulting values are around a mean value of 2.5, with a range of 0.5 to 5.7, depending on the gripper version, and the dimensions and orientation of the test object. The low to moderate standard deviation values indicate good repeatability and confirm that the experiments were conducted in a consistent and methodologically sound manner. Detailed standard deviation values are provided in Supplementary Materials (Table S1).

Four test objects of different shapes and dimensions were selected for the experiments. Their geometrical and physical characteristics are summarized in Table 2. The first two test objects were cylindrical hex socket wrench heads of different diameters (WH12 and WH20), selected to represent realistic industrial components, as illustrated in Figure 1c. These steel components feature smooth surfaces, posing an additional challenge for achieving a reliable and stable grasp. In addition, measurements were conducted using two wooden spheres (SP27 and SP37), allowing analysis of gripper behavior under different friction conditions compared to steel objects. The hex socket wrench heads were tested in vertical and horizontal orientations relative to the gripper axis to evaluate the effect of object orientation on grasping performance, whereas the spherical objects were tested in a single orientation due to their geometric symmetry.

**Figure 4:**
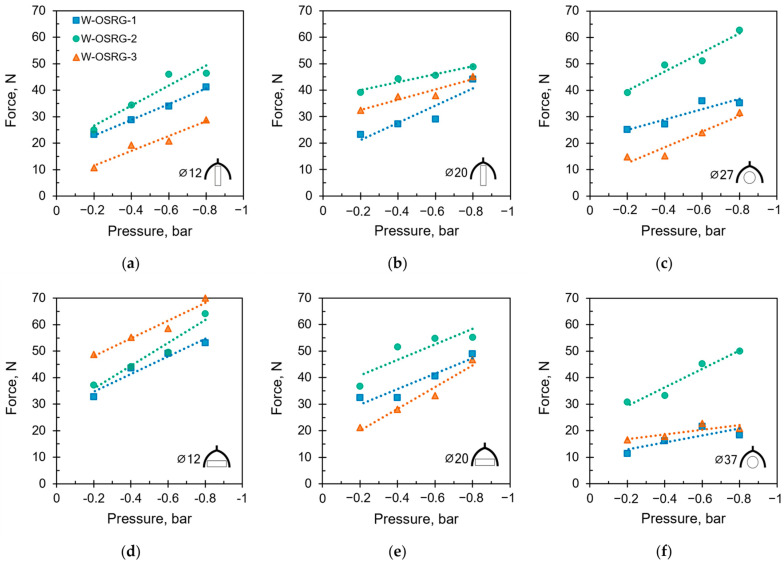
Holding forces of the W-OSRG for different test objects and orientations: (**a**) WH12—vertical; (**b**) WH20—vertical; (**c**) S27; (**d**) WH12—horizontal; (**e**) WH20—horizontal; (**f**) S37.

The first experimental measurements were performed on the developed W-OSRG across all three variations. The results, shown in Figure 4, clearly demonstrate that increasing negative pressure leads to an increase in holding force. This behavior is consistent with Pascal’s law and was observed throughout all subsequent experiments. The obtained results indicate that W-OSRG-2 consistently achieves higher holding forces than W-OSRG-1 due to the larger surface area of the origami skeleton exposed to negative pressure (*F* = 64 N at *p* = −0.8 bar). Furthermore, the paper-based W-OSRG-2 achieved higher holding forces than the polymer-laminated W-OSRG-3, except in the case of WH12 in the horizontal orientation. The reduced holding forces of W-OSRG-3 are attributed to deformation of the polymer-laminated paper skeleton, which stores higher strain energy and thereby increases resistance during grasping.

Comparison of the results for WH12 and WH20 in vertical and horizontal orientations reveals higher grasping forces in the horizontal orientation due to more effective closure of the external and internal teeth around the objects. As noted, W-OSRG-2 demonstrated the highest holding forces for most tested objects; however, W-OSRG-3 achieved the maximum holding force of *F* = 70 N for WH12 in the horizontal orientation at *p* = −0.8 bar (Figure 4d). This behavior is attributed to deformation of the paper skeletons under elevated holding forces, which promotes slippage of the test object from the skeleton teeth, whereas the polymer-laminated paper skeleton of W-OSRG-3 exhibits reduced deformation and consequently higher grasping forces. On the other hand, in the vertical orientation, W-OSRG-3 exhibits lower performance than W-OSRG-1 and W-OSRG-2 (Figure 4a) due to the reduced radial contraction of the polymer-laminated skeleton, which increases the minimum closing gap between teeth and makes smaller, narrower objects more prone to slippage. Experimental measurements conducted with S27 and S37 confirm that W-OSRG-2 achieves the highest holding force for S27, reaching *F* = 62.8 N at *p* = −0.8 bar, whereas the comparable force for S37 is *F* = 50 N (Figure 4c,f). W-OSRG-3 exhibited significantly lower performance for S27 due to the insufficient deformability of the polymer-laminated skeleton, which limited firm contact with smaller spherical objects.

**Figure 5:**
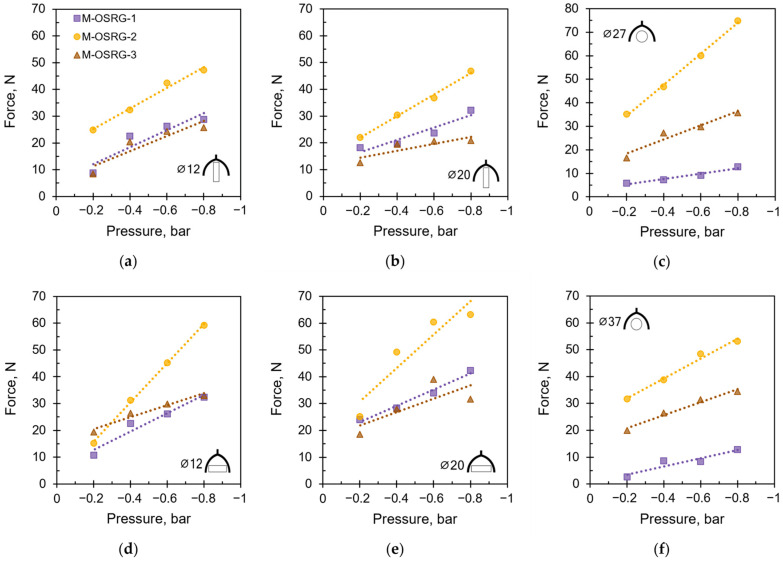
Holding forces of the M-OSRG for different test objects and orientations: (**a**) WH12—vertical; (**b**) WH20—vertical; (**c**) S27; (**d**) WH12—horizontal; (**e**) WH20—horizontal; (**f**) S37.

The second set of experimental measurements was performed on the developed M-OSRG across all three versions (Figure 5). The results achieved indicate that M-OSRG-2 consistently exhibits the highest holding forces across all test objects and orientations considered. Again, this enhanced performance can be attributed to the larger surface area of the paper origami skeleton and the paper’s lower stiffness. In addition, M-OSRG-1 achieves higher holding forces than M-OSRG-3 when grasping cylindrical objects, WH12 and WH20, despite the larger skeleton dimensions of M-OSRG-3 (Figure 5a,b,e). These findings further suggest that an increase in the stiffness of the skeleton material negatively affects grasping efficiency. Orientation-dependent analysis indicates that WH12 and WH20 achieve higher holding forces in the horizontal orientation due to improved contact between the gripper teeth and the object. The highest force was obtained in the horizontal orientation with M-OSRG-2, reaching *F* = 63.2 N at *p* = −0.8 bar (Figure 5e).

Furthermore, experimental measurements conducted with wooden spheres show that M-OSRG-2 achieves the highest holding forces (Figure 5c), with a maximum of *F* = 74.8 N recorded for S27 at *p* = −0.8 bar, compared to *F* = 53.2 N for S37. These results indicate that the geometry of the gripper teeth enables the M-OSRG-2 to adapt more effectively to smaller spherical objects. In contrast, for M-OSRG-1, nearly identical holding forces were obtained for S27 and S37, with maximum values remaining around 10 N, which can be attributed to the smaller skeleton dimensions that limit effective engagement of the internal teeth and consequently reduce grasping capability for spherical objects. Moreover, the higher holding forces achieved by M-OSRG-3 compared to M-OSRG-1 can be attributed to the reduced deformation of the polymer-laminated paper skeleton during pulling of the test object.

To evaluate the effect of increased origami skeleton dimensions on grasping performance, the holding forces of OSRG-1 and OSRG-2 were compared for both developed grippers. Table S2 in Supplementary Materials summarizes the average holding forces and corresponding force increase factors for each test object and negative-pressure value. Analysis of the measured results for the paper-based OSRG-1 and OSRG-2 indicates that increasing the number of columns (*n*) enhances the holding force, with the largest improvements observed for the smaller test objects WH12 and S27. Particularly notable is the M-OSRG-1, which achieved a 12-fold increase in holding force for S27 and S37 at *p* = −0.2 bar, representing the largest improvement among all tested grippers and objects.

Furthermore, a comparison of the average holding forces was conducted for the developed OSRGs, with the results systematically classified according to origami pattern and material type to enable a detailed quantitative analysis of the individual and combined effects of structural geometry and material characteristics on gripper performance. Figure S1 in Supplementary Materials presents the experimental results for OSRG-1, showing that W-OSRG-1 consistently achieved the highest holding forces for the cylindrical test objects WH12 and WH20 in both orientations, while also exhibiting stable performance when grasping the spherical objects S27 and S37. These results demonstrate that the W-OSRG-1 provides the most effective two-stage grasping, making it well-suited for stable manipulation of cylindrical and spherical objects. The M-OSRG-1 exhibits good performance when grasping cylindrical test objects WH12 and WH20 but shows substantially reduced effectiveness for spherical objects S27 and S37, rendering it overall less effective than the W-OSRG-1.

Results obtained using the proposed energy-based model demonstrate that the model successfully captures the nonlinear relationship between negative pressure and holding force, reproducing the dominant trend of increasing holding force with increasing negative pressure and showing good agreement with the experimental measurements. Model validation was performed using the spherical test objects S27 and S37 due to their symmetric geometry, which simplifies penetration depth analysis during grasping. The modeled results are presented in Figure S1c,f as red square markers for W-OSRG-1 and brown square markers for M-OSRG-1. These findings confirm that the proposed energy-based model provides a physically interpretable reduced-order representation of the dominant pressure–force behavior of the origami-based soft robotic gripper system.

Figure S2 in Supplementary Materialspresents the experimental results for OSRG-2, showing that W-OSRG-2 maintains consistently strong performance, particularly for the cylindrical test object WH12. For WH20, W-OSRG-2 achieves higher holding forces in the vertical orientation, whereas in the horizontal orientation and at pressures above *p* = −0.5 bar, M-OSRG-2 exhibits better performance due to more effective grasping by the external gripper teeth. When grasping spherical objects, M-OSRG-2 exhibits slightly notable performance, particularly for SP27, where the maximum holding force was achieved (*F* = 74.8 N at *p* = −0.8 bar), as shown at Figure S2c. Similar agreement between the experimental measurements and the proposed energy-based model was also obtained for the W-OSRG-2 (purple dot) and M-OSRG-2 (blue dot). The modeled results successfully reproduce the nonlinear increase in holding force with increasing negative pressure, confirming that the proposed reduced-order formulation captures the dominant pressure–force behavior of the developed origami-based soft robotic grippers.

Experimental results obtained for polymer-laminated paper OSRG-3, presented in Figure S3 in Supplementary Materials, again demonstrate that W-OSRG-3 achieves superior performance compared to M-OSRG-3, particularly for cylindrical test objects. The most pronounced increase in holding force is observed during grasping of WH12 in the horizontal orientation, where the internal teeth engage the object more effectively, resulting in enhanced grasping performance. Comparison of the results obtained for spherical test objects reveals that M-OSRG-3 achieves slightly better performance than W-OSRG-3, further confirming the improved grasping capability of the M-OSRG for spherical objects. Similar agreement between the experimental and modeled results was also obtained for W-OSRG-3 (black triangles) and M-OSRG-3 (green triangles), confirming that the proposed energy-based model successfully captures the dominant nonlinear pressure–force behavior of the developed grippers.

Furthermore, the normalized holding-force performance of the developed origami-based grippers is summarized in Table 3. The calculated force-to-weight ratios range from 141.3 to 346.6, indicating that the developed grippers are capable of generating holding forces more than two orders of magnitude greater than their own weight. Among the waterbomb-based configurations, W-OSRG-2 achieved the highest force-to-weight ratio (327.2), whereas W-OSRG-3 exhibited a lower value (237.8). This reduction can be attributed to the increased mass of the polymer-laminated skeleton, which outweighed the corresponding improvement in grasping performance. A similar trend was observed for the Miura-ori grippers, where M-OSRG-2 achieved the highest normalized performance (346.6), while M-OSRG-3 showed the lowest value (141.3). Overall, the results indicate that increasing the dimensions of the origami skeleton generally improves the normalized grasping performance; however, additional reinforcement through skeleton lamination increases the gripper mass and may reduce the force-to-weight ratio despite improving structural durability.

The developed OSRGs can be positioned within the broader field of biomimetic and origami-based soft robotic grippers. Previous studies have demonstrated the potential of origami-inspired designs for adaptive grasping through various mechanisms, including variable effective-length structures [28], graded Miura-ori architectures [29], tunable constant-force grasping [30], and vacuum-driven Magic-Ball configurations [31]. Additional soft gripper concepts have employed origami-based enclosing grasping [19], insect-inspired claw mechanisms [20], and stiffness-tunable structures with enhanced load-carrying capacity [21].

In contrast, the proposed OSRGs introduce a biomimetic two-stage grasping mechanism inspired by the sea urchin mouth, integrating external and internal teeth within modified waterbomb and Miura-ori origami architectures. Unlike most existing origami-based grippers that rely on single-stage grasping, our novel designs enable two-stage grasping, combining lightweight, low-cost fabrication with experimentally validated results. The experimental results demonstrated holding forces of up to 70 N and 74.8 N for the waterbomb and Miura-ori architectures, respectively, together with force-to-weight ratios reaching 327.2 and 346.6. These findings highlight the potential of the proposed architectures for achieving high load-carrying capability while maintaining the inherent advantages of lightweight origami-based construction.

Despite the promising results, several limitations of the present study should be acknowledged. First, the experiments were conducted under controlled laboratory conditions; therefore, the performance of the developed grippers in more realistic environments remains to be investigated. Second, the experimental evaluation was performed using a limited set of test objects, and additional studies involving a broader range of object geometries, sizes, and surface characteristics would provide a more comprehensive assessment of grasping performance. Furthermore, the grippers were fabricated manually, which may introduce manufacturing variability between individual prototypes. Finally, the long-term durability of the origami skeletons, including fatigue under repeated actuation cycles and the durability of the balloon skeleton interface, was not investigated. These aspects will be addressed in future work to further evaluate the robustness and practical applicability of the developed grippers.

## 4. Conclusions

In this study, we presented an innovative origami-based soft robotic gripper capable of two-stage grasping. The proposed design introduces a novel biomimetic interpretation of the sea urchin mouth by integrating the functions of external rigid teeth and an internal soft peristomial membrane into a soft robotic grasping system. To realize this concept, a negative-pressure-actuated origami-based gripper was developed by combining an origami skeleton with a compliant outer membrane, enabling lightweight, low-cost, and structurally adaptive grasping. Waterbomb and Miura-ori origami patterns were selected for the gripper skeleton due to their ability to form two distinct segments corresponding to the external and internal teeth required for two-stage grasping. Modified waterbomb and Miura-ori origami patterns were introduced, where a half-height unit cell forms the external teeth, a standard cell generates the internal teeth, and the folded upper section creates a conical geometry that enhances geometric adaptability and kinematic performance. In addition, the Miura-ori architecture generates a slightly conical inner skeleton geometry, where initial contact is established by the internal teeth and subsequently reinforced by the external teeth. Multiple gripper configurations with varying dimensions were developed using both paper and polymer-laminated paper origami skeletons. We also presented an energy-based model capturing the dominant pressure–force behavior of the developed grippers and enabling qualitative and semi-quantitative comparison with experimental results. Experimental evaluations conducted at different negative-pressure values demonstrated reliable grasping of delicate and irregularly shaped objects, while imprint analysis on clay test objects confirmed the proposed two-stage grasping mechanism. Experimental holding force evaluations were conducted using cylindrical and spherical test objects, including both vertical and horizontal orientations for cylindrical objects, revealing that the waterbomb-based gripper exhibits superior performance for cylindrical objects, whereas the Miura-ori configuration provides improved adaptability and higher holding forces for spherical objects. The normalized performance analysis demonstrated that the developed grippers can generate holding forces up to 346 times greater than their own weight, highlighting their high load-carrying capability.

The results obtained in this study demonstrate the effectiveness of the developed paper-based and polymer-laminated paper origami grippers, while highlighting material-related limitations concerning long-term durability and fatigue resistance. Future work will therefore focus on the development of origami skeletons fabricated using resin-based 3D printing technologies, as well as on the integration and validation of the proposed grippers on an industrial robotic manipulator in real-world grasping applications.

## Figures and Tables

**Table 1:** Effective axial and circumferential projections of a single origami unit cell.

**Origami Pattern**	$l_{z}(\theta )$	$l_{c}(\theta )$
Waterbomb	acosα cosθ	asinα sinθ
Miura-ori	$a\sqrt{1-{sin}^{2}\alpha \;{sin}^{2}\theta }$	acosθ

**Table 2:** Characteristics of the test objects used in the experimental evaluation.

Test Object	Shape	Material	Diameter, mm	Length, mm	Mass, g
CL1	Cylindrical	Modeling clay	25	45	30
CL2	Spherical	Modeling clay	35	-	30
Plastic cup	Irregular	Plastic	70/43	85	3
Cherry tomato	Irregular	Biological	~30	-	15
Lemon	Irregular	Biological	~40	~50	50
Pliers	Irregular	Steel	-	170	180
Throttle check valve	Irregular	Plastic	-	40	10
Angle bracket	Irregular	Aluminum	-	27/27	25
WH12	Cylindrical	Steel	12	25	11
WH20	Cylindrical	Steel	20	25	34
S27	Spherical	Wood	27	-	5
S37	Spherical	Wood	37	-	11

**Table 3:** Maximum holding force and force-to-weight ratio of the developed OSGRs.

OSRG	Mass, g	Max. Holding Force, N	Force-to-Weight Ratio
W-OSRG-1	18.5	53.2	293.1
W-OSRG-2	20	64.2	327.2
W-OSRG-3	30	70	237.8
M-OSRG-1	21	42.4	205.8
M-OSRG-2	22	74.8	346.6
M-OSRG-3	28	35.8	141.3

## Data Availability

Data is contained within the article or Supplementary Materials.

## Supplementary Materials

The following supporting information can be downloaded at https://www.mdpi.com/article/10.3390/biomimetics11070466/s1: Video S1: Grasping capability of developed origami-based soft robotic grippers; Table S1: Standard deviation of the experimental holding force measurements; Table S2: Holding forces and force increase factors associated with increased skeleton dimensions; Figure S1: Holding forces of the OSRG-1 for different test objects and orientations: (a) WH12—vertical; (b) WH20—vertical; (c) S27; (d) WH12—horizontal; (e) WH20—horizontal; (f) S37; Figure S2: Holding forces of the OSRG-2 for different test objects and orientations: (a) WH12—vertical; (b) WH20—vertical; (c) S27; (d) WH12—horizontal; (e) WH20—horizontal; (f) S37; Figure S3: Holding forces of the OSRG-3 for different test objects and orientations: (a) WH12—vertical; (b) WH20—vertical; (c) S27; (d) WH12—horizontal; (e) WH20—horizontal; (f) S37.

## Abbreviations

The following abbreviations are used in this manuscript:

TPU	Thermoplastic polyurethane
PLA	Polylactic acid
PET	Polyethylene terephthalate
PVC	Polyvinyl chloride
OSRG	Origami-based soft robotic gripper
OSRG-1	Origami-based soft robotic gripper—Version 1 with a paper skeleton and origami pattern columns (*n* = 8)
OSRG-2	Origami-based soft robotic gripper—Version 2 with a paper skeleton and origami pattern columns (*n* = 10)
OSRG-3	Origami-based soft robotic gripper—Version 3 with a polymer-laminated paper skeleton and origami pattern columns (*n* = 10)
W-OSRG	Waterbomb origami-based soft robotic gripper
M-OSRG	Miura-ori origami-based soft robotic gripper
W-OSRG-1	Waterbomb origami-based soft robotic gripper—Version 1 with a paper skeleton and origami pattern columns (*n* = 8)
W-OSRG-2	Waterbomb origami-based soft robotic gripper—Version 2 with a paper skeleton and origami pattern columns (*n* = 10)
W-OSRG-3	Waterbomb origami-based soft robotic gripper—Version 3 with a polymer-laminated paper skeleton and origami pattern columns (*n* = 10)
M-OSRG-1	Miura-ori origami-based soft robotic gripper—Version 1 with a paper skeleton and origami pattern columns (*n* = 8)
M-OSRG-2	Miura-ori origami-based soft robotic gripper—Version 2 with a paper skeleton and origami pattern columns (*n* = 10)
M-OSRG-3	Miura-ori origami-based soft robotic gripper—Version 3 with a polymer-laminated paper skeleton and origami pattern columns (*n* = 10)
CL1	Cylindrical modelling clay test object
CL2	Spherical modelling clay test object
WH12	Cylindrical test objects—hex socket wrench head (diameter = 12 mm)
WH20	Cylindrical test objects—hex socket wrench head (diameter = 20 mm)
SP27	Spherical test objects—wooden sphere (diameter = 27 mm)
SP37	Spherical test objects—wooden sphere (diameter = 37 mm)

## References

[1] Kasahara K. (1998). Origami Omnibus: Paper-Folding for Everybody.

[2] Song Z., Zhu J.F., Wang X., Zhang R., Min P., Cao W., He Y., Han J., Wang T., Zhu J. (2024). Origami metamaterials for ultra-wideband and large-depth reflection modulation. Nat. Commun..

[3] Ahmed A.R., Gauntlett O.C., Camci-Unal G. (2020). Origami-inspired approaches for biomedical applications. ACS Omega.

[4] Joshi M. (2022). Creative Use of Industrial Waste into Fashion Products through Fabric Manipulation Using Origami Technique. ECS Trans..

[5] Ebrahimi Fakhari H., Rosario Barboza J., Mardanpour P. (2024). Biomimetic Origami: A Biological Influence in Design. Biomimetics.

[6] Rus D., Tolley M.T. (2015). Design, fabrication and control of soft robots. Nature.

[7] Ren T., Chen Y., Li Y., Yang Y., Chen Y., Yang S.X., Li Y. (2025). Mathematical evolution of origami structures and their applications in soft robotics. IEEE Trans. Autom. Sci. Eng..

[8] Eyvazian A., Song Y., Hovhannes C., Savari A., Singh N.S.S. (2026). State-of-the-art soft robotic systems for unstructured and real-world environments: A systematic review. Eng. Sci. Technol. Int. J..

[9] Rajappan A., Jumet B., Preston D.J. (2021). Pneumatic soft robots take a step toward autonomy. Sci. Robot..

[10] Gunawardane P.D.S.H., Cheung P., Zhou H., Alici G., de Silva C.W., Chiao M. (2024). A versatile 3D-printable soft pneumatic actuator design for multi-functional applications in soft robotics. Soft Robot..

[11] Mak Y.X., Naghibi H., Lin Y., Abayazid M. (2023). Adaptive control of a soft pneumatic actuator using experimental characterization data. Front. Robot. AI.

[12] Xavier M.S., Tawk C.D., Yong Y.K., Fleming A.J. (2021). 3D-printed omnidirectional soft pneumatic actuators: Design, modeling and characterization. Sens. Actuators A Phys..

[13] Bazina T., Kladarić M., Kamenar E., Gregov G. (2024). Development of Rehabilitation Glove: Soft Robot Approach. Actuators.

[14] Xiao W., Xie C., Xiao Y., Tang K., Wang Z., Hu D., Ding R., Jiao Z. (2025). A new vacuum-powered soft bending actuator with programmable variable curvatures. Mater. Des..

[15] Lee J.G., Rodrigue H. (2019). Origami-based vacuum pneumatic artificial muscles with large contraction ratios. Soft Robot..

[16] Gregov G., Vuković T., Gašparić L., Pongrac M. (2025). Development, experimental assessment, and application of a vacuum-driven soft bending actuator. Appl. Sci..

[17] AboZaid Y.A., Aboelrayat M.T., Fahim I.S., Radwan A.G. (2024). Soft robotic grippers: A review on technologies, materials, and applications. Sens. Actuators A Phys..

[18] Zhang Y., Zhang W., Gao P., Zhong X., Pu W. (2022). Finger-palm synergistic soft gripper for dynamic capture via energy harvesting and dissipation. Nat. Commun..

[19] Liu J., Chen Z., Wen G., He J., Wang H., Xue L., Long K., Xie Y.M. (2023). Origami chomper-based flexible gripper with superior gripping performances. Adv. Intell. Syst..

[20] Yang L., Miao J., Li G., Ren H., Zhang T., Guo D., Tang Y., Shang W., Shen Y. (2022). Soft Tunable Gelatin Robot with Insect-like Claw for Grasping, Transportation, and Delivery. ACS Appl. Polym. Mater..

[21] Hu T., Lu X., Xu D. (2023). A dual-mode and enclosing soft robotic gripper with stiffness-tunable and high-load capacity. Sens. Actuators A Phys..

[22] Liu J., Zhu Z., Wen L. (2026). Underwater soft arm grasping with simplified control using octopus-inspired bending propagation. npj Robot..

[23] Liu L., Zhang J., Liu G., Zhu Z., Hu Q., Li P. (2022). Three-fingered soft pneumatic gripper integrating joint-tuning capability. Soft Robot..

[24] Li S., Vogt D.M., Rus D., Wood R.J. (2017). Fluid-driven origami-inspired artificial muscles. Proc. Natl. Acad. Sci. USA.

[25] Khosravi H., Iannucci S.M., Li S. (2021). Pneumatic soft actuators with kirigami skins. Front. Robot. AI.

[26] Chen C., Liu Z., Shi P., Zhao Y., Duan S., Du Y., Yan Y., Si M., Iwasaki T., He X. (2025). Bio-inspired multimodal soft actuator with environmental self-adaptation. Nat. Commun..

[27] Niu M., Hu X., Yuan C., Li H., Su X., Sun F. (2026). Bioinspired soft-skeleton robotics with cooperative shape transformation and stiffness adaptation. Adv. Robot. Res..

[28] Chen B., Shao Z., Xie Z., Liu J., Pan F., He L., Zhang L., Zhang Y., Ling X., Peng F. (2021). Soft origami gripper with variable effective length. Adv. Intell. Syst..

[29] Fang Q.Y., Xu S.F., Chu M.S., Yan T., Xu Z.L., Wu T.Y., Wang D.F., Tachi T., Chuang K.C. (2024). Graded in-plane Miura origami as crawling robots and grippers. J. Appl. Phys..

[30] Ni Z., Xu C., Qin Z., Zhang C., Tang Z., Wang P., Laschi C. (2025). Origami-inspired soft gripper with tunable constant force output. Proceedings of the 2025 IEEE/RSJ International Conference on Intelligent Robots and Systems (IROS).

[31] Li S., Stampfli J.J., Xu H.J., Malkin E., Diaz E.V., Rus D., Wood R.J. (2019). A vacuum-driven origami “magic-ball” soft gripper. Proceedings of the 2019 International Conference on Robotics and Automation (ICRA).

[32] Stiefel K.M., Barrett G.A. (2018). Sea urchins as an inspiration for robotic designs. J. Mar. Sci. Eng..

[33] Frank M.B., Naleway S.E., Wirth T.S., Jung J.-Y., Cheung C.L., Loera F.B., Medina S., Sato K.N., Taylor J.R.A., McKittrick J. (2016). A protocol for bioinspired design: A ground sampler based on sea urchin jaws. J. Vis. Exp..

[34] Ma J., Feng H., Chen Y., Hou D., You Z. (2020). Folding of tubular waterbomb. Research.

