# Supplementary material for: Biomimetic Origami-Based Soft Robotic Grippers with Two-Stage Grasping
Source: Biomimetics (Basel). 2026 Jul 3;11(7):466. doi: 10.3390/biomimetics11070466 (PMC13406422; doi:10.3390/biomimetics11070466)
Supplement: Supplementary file 1 [file biomimetics-11-00466-s001.zip › Supplementary materials.pdf]

Table S1. Standard deviation of the experimental holding force measurements

OSRG	Pressure [bar]	WH12 vertical	WH12 horizontal	WH20 vertical	WH20 horizontal	S27	S37
**W-OSRG-1**	-0.2	2.2804	3.3466	3.033	2.6077	4.1473	2.6077
	-0.4	2.2804	3.2863	2.280	1.6733	2.2804	3.0332
	-0.6	3.1623	2.2804	1	1.9494	3.1623	2.6077
	-0.8	2.2804	3.0332	1.483	2	3.0332	1.6733
**W-OSRG-2**	-0.2	3.4641	1.7889	3.0332	1.0954	1.0954	1.0954
	-0.4	2.6077	1.3038	3.2863	0.8944	1.6733	1.0954
	-0.6	2.4495	1.6733	2.9665	1.7889	5.7184	1.0954
	-0.8	3.8471	1.4832	1.0954	2.2804	1.7889	2
**W-OSRG-3**	-0.2	1.7889	2.2804	2.6077	2.2804	3.6332	3.1305
	-0.4	3.0332	2.2804	1.6733	3.1623	2.2804	1.4832
	-0.6	2.2804	5.1769	2	2.2804	3.1623	3.0332
	-0.8	3.0332	1.9494	2.2804	3.6332	1.6733	3.3466
**M-OSRG-1**	-0.2	2.2804	2.2804	2.2804	3.1623	1.4832	0.5477
	-0.4	2.4083	2.4083	1.6733	2.6077	1.7889	0.8944
	-0.6	2.8636	2.8636	1.1402	1.4142	1.7889	1.6733
	-0.8	1.6432	1.6733	2.2804	1.6733	2.1679	2.1679
**M-OSRG-2**	-0.2	5.7619	2.2804	2	2.2804	1.0954	1.6733
	-0.4	3.8471	4.6043	1.6733	1.0954	1.0954	1.0954
	-0.6	2.6077	2.2804	1.0954	2.6077	1.6733	1.6733
	-0.8	1.0954	5.7619	1.0954	1.0954	3.6332	1.0954
**M-OSRG-3**	-0.2	2.6077	4.7749	3.9749	0.8944	0.8944	2.4495
	-0.4	4.3932	1.6733	1.3416	1.4142	2.3875	1.6733
	-0.6	5.6833	3.6332	2.1909	5.0990	2.6833	3.4351
	-0.8	5.9498	2.9155	2.2804	2.7019	1.7889	1.6733

Table S2. Holding forces and force increase factors associated with increased skeleton dimensions

	Test object	Pressure [bar]	Holding force [N]		Force increase factor
			OSRG-1	OSRG-2	
W-OSRG	WH12 vertical	-0.2	23.2	25	1.08
		-0.4	28.8	34.4	1.19
		-0.6	34	46	1.35
		-0.8	41.2	46.4	1.13
	WH12 horizontal	-0.2	32.8	37.2	1.13
		-0.4	43.6	44.2	1.01
		-0.6	49.2	49.6	1.01
		-0.8	53.2	64.2	1.21
	WH20 vertical	-0.2	23.2	39.2	1.69
		-0.4	27.2	44.4	1.63
		-0.6	29	45.6	1.57
		-0.8	44.2	48.8	1.10
	WH20 horizontal	-0.2	32.4	36.8	1.14
		-0.4	32.4	51.6	1.59
		-0.6	40.6	54.8	1.35
		-0.8	49	55.2	1.13
	S27	-0.2	25.2	39.2	1.56
		-0.4	27.2	49.6	1.82
		-0.6	36	51.2	1.42
		-0.8	35.2	62.8	1.78
	S37	-0.2	11.4	30.8	2.70
		-0.4	16.2	33.2	2.05
		-0.6	21.6	45.2	2.09
		-0.8	18.4	50	2.72
M-OSRG	WH12 vertical	-0.2	8.8	24.8	2.82
		-0.4	22.6	32.4	1.43
		-0.6	26.2	42.4	1.62
		-0.8	28.8	47.2	1.64
	WH12 horizontal	-0.2	10.8	15.2	1.41
		-0.4	22.6	31.2	1.38
		-0.6	26.2	45.2	1.73
		-0.8	32.4	59.2	1.83
	WH20 vertical	-0.2	18.2	22	1.21
		-0.4	19.6	30.4	1.55
		-0.6	23.6	36.8	1.56
		-0.8	32.2	46.8	1.45
	WH20 horizontal	-0.2	24	25.2	1.05
		-0.4	28.4	49.2	1.73
		-0.6	34	60.4	1.78
		-0.8	42.4	63.2	1.49
	S27	-0.2	5.8	35.2	6.07
		-0.4	7.2	46.8	6.50
		-0.6	9.2	60	6.52
		-0.8	12.8	74.8	5.84
	S37	-0.2	2.6	31.6	12.15
		-0.4	8.6	38.8	4.51
		-0.6	8.4	48.4	5.76
		-0.8	12.8	53.2	4.16

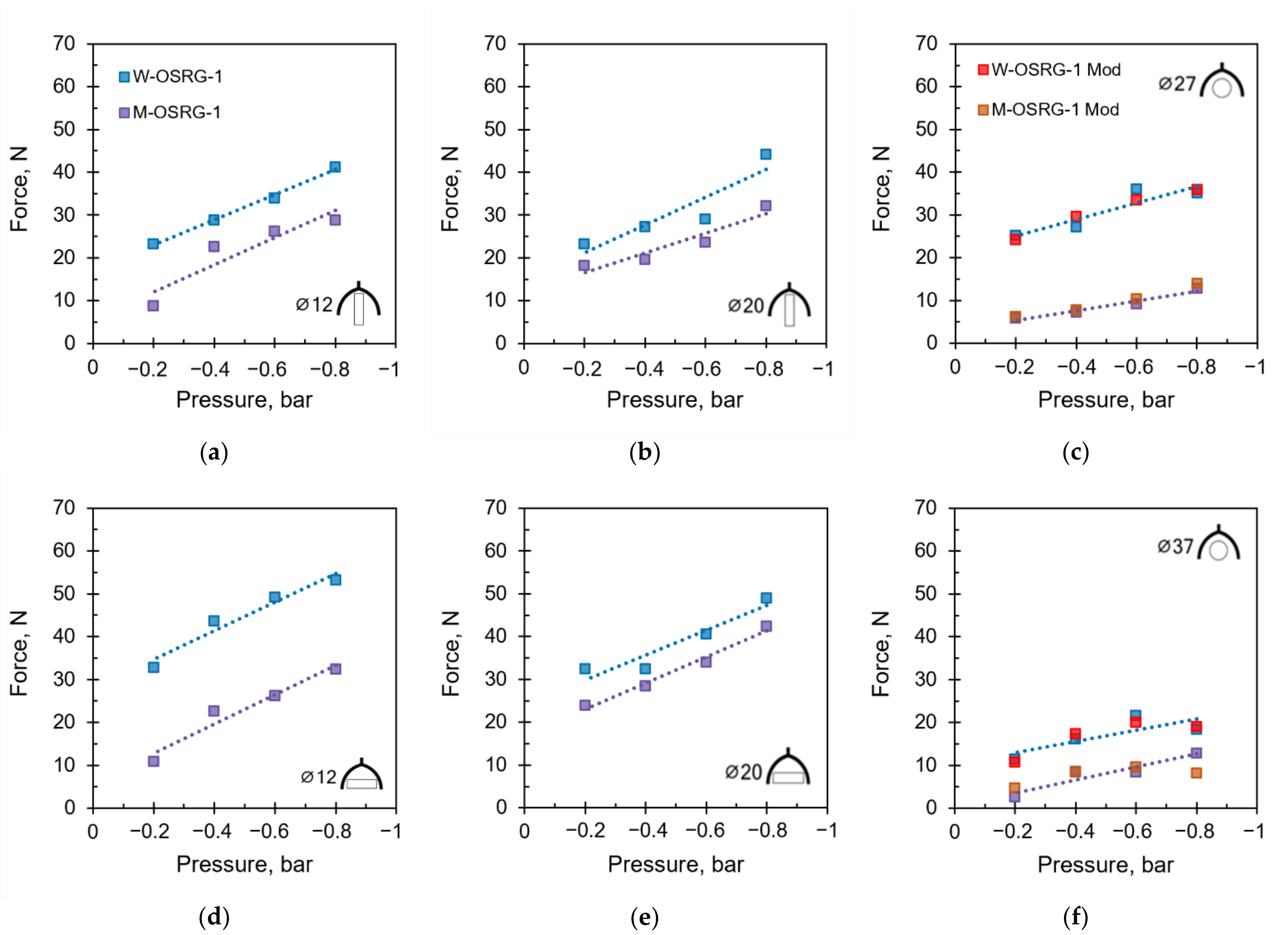

Figure S1. Holding forces of the OSRG-1 for different test objects and orientations: (**a**) WH12-vertical; (**b**) WH20-vertical; (**c**) S27; (d) WH12-horizotal; (e) WH20-horizotal; (f) S37.

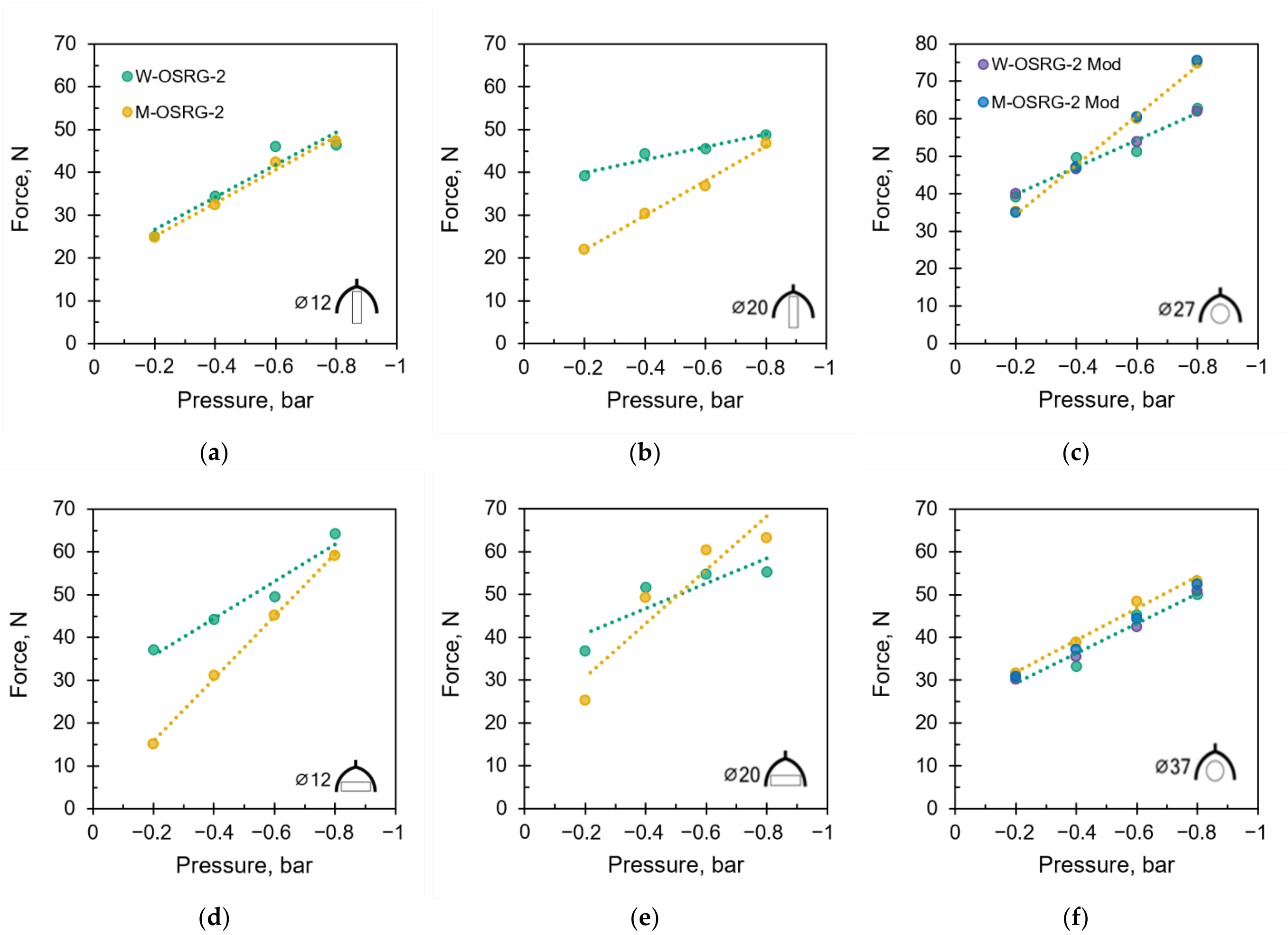

Figure S2. Holding forces of the OSRG-2 for different test objects and orientations: (**a**) WH12-vertical; (**b**) WH20-vertical; (**c**) S27; (d) WH12-horizotal; (e) WH20-horizotal; (f) S37.

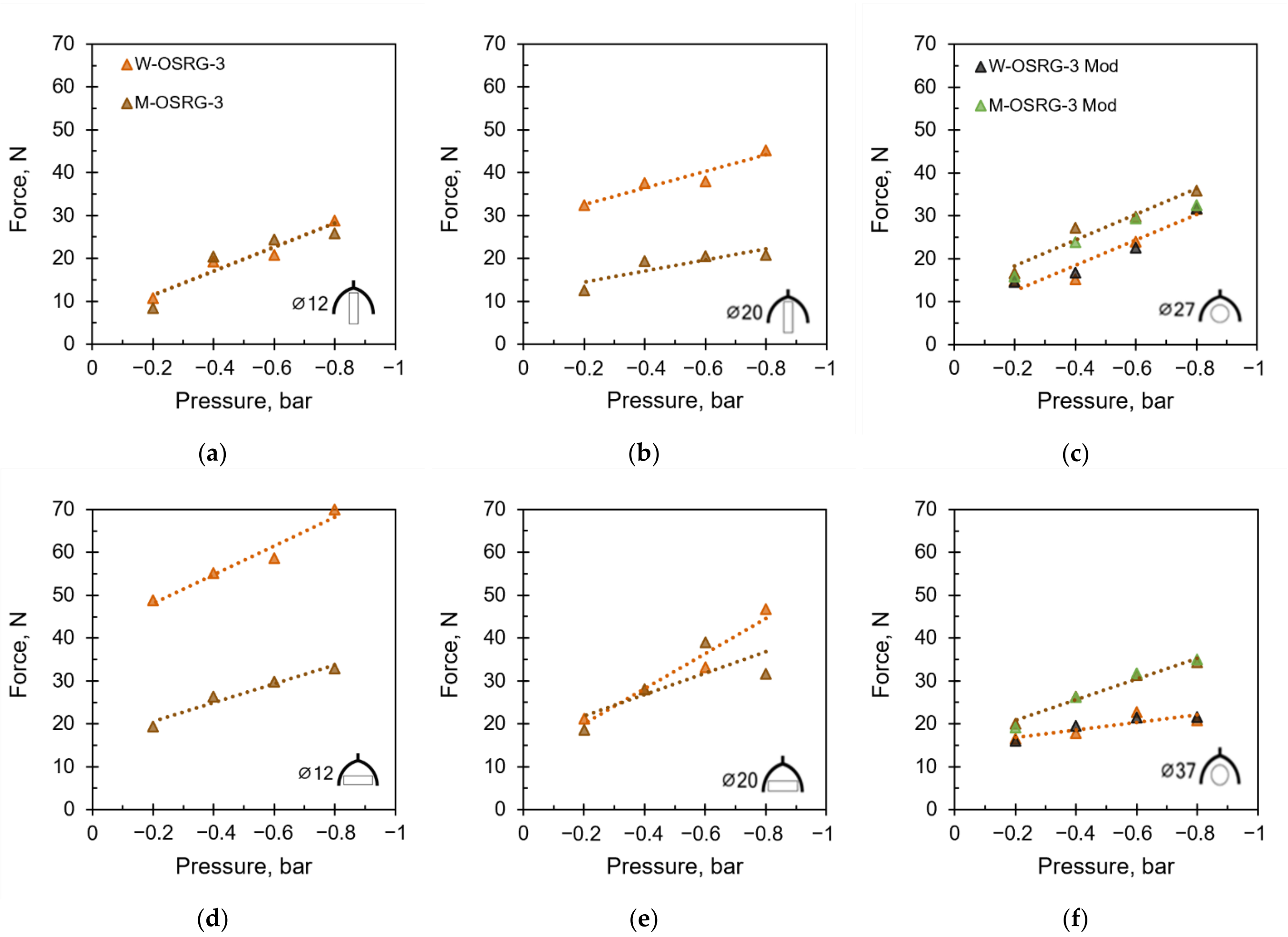

Figure S3. Holding forces of the OSRG-3 for different test objects and orientations: (**a**) WH12-vertical; (**b**) WH20-vertical; (**c**) S27; (d) WH12-horizotal; (e) WH20-horizotal; (f) S37.
